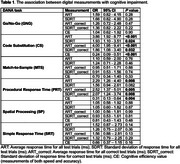# Effectiveness of Self‐Administered Mobile Assessment in Detecting Mild Cognitive Impairment

**DOI:** 10.1002/alz70856_105199

**Published:** 2026-01-07

**Authors:** Huitong Ding, Chenglin Lyu, Edward Searls, Kristi Ho, Zexu Li, Alexa Burk, Margaret Low, Kaitlyn Anderson, Owen Tan, Xavier Serrano, Eric G. Steinberg, Jesse Mez, Katherine A. Gifford, Michael L Alosco, Vijaya B. Kolachalama, Honghuang Lin, Rhoda Au

**Affiliations:** ^1^ Boston University Chobanian & Avedisian School of Medicine, Boston, MA, USA; ^2^ Framingham Heart Study, Framingham, MA, USA; ^3^ Dept of Anatomy & Neurobiology, Boston University Chobanian & Avedisian School of Medicine, Boston, MA, USA; ^4^ Boston University Alzheimer's Disease Research Center & CTE Center, Boston, MA, USA; ^5^ Boston University Chronic Traumatic Encephalopathy Center, Boston, MA, USA; ^6^ Boston University Alzheimer's Disease Research Center, Boston, MA, USA; ^7^ Framingham Heart Study, Boston University Chobanian & Avedisian School of Medicine, Boston, MA, USA; ^8^ Computing & Data Sciences, Boston University, Boston, MA, USA; ^9^ University of Massachusetts Chan Medical School, Worcester, MA, USA; ^10^ Boston University Alzheimer's Disease Research Center, Boston University Chobanian & Avedisian School of Medicine, Boston, MA, USA; ^11^ Boston University Chobanian & Avedisian School of Medicine and School of Public Health, Boston, MA, USA

## Abstract

**Background:**

Self‐administered mobile cognitive assessment tools such as the Defense Automated Neurobehavioral Assessment (DANA) have recently emerged as promising solutions for the efficient monitoring of cognitive health. This study investigated the association of DANA with the risk of mild cognitive impairment (MCI).

**Method:**

The study sample included participants enrolled in the Boston University Alzheimer's Disease Research Center (BU ADRC) who completed six DANA tasks on their smartphone, yielding five digital cognitive measures per task (four response time metrics and cognitive efficiency). Participants were categorized as either cognitively intact or diagnosed with MCI based on consensus diagnostic meetings at the BU ADRC, adhering to the criteria set by the National Alzheimer's Coordinating Center Uniform Data Set. Digital measures were standardized to have a mean of zero and a standard deviation of one. Logistic regression analyses, adjusted for age, sex, and education, related digital cognitive measures to cognitive status.

**Result:**

A total of 132 participants were included in the study (mean age 71.9 ± 10.2 years, 57.6% female), among which, 17 were diagnosed as MCI. All five digital measurements from the code substitution task were associated with MCI. Each standard deviation increase in cognitive efficiency in the code substitution task was associated with a 76% reduction in the odds of MCI (OR = 0.24, 95% CI = 0.09‐0.51, *P* < 0.001). All except standard deviation of response time for all test trials from the procedural response time task were associated with MCI. However, none of the digital measurements from the go/no‐go task, match‐to‐sample, spatial processing, and simple response time were associated with MCI.

**Conclusion:**

Digital measures associated with executive function appear to be most sensitive to the identification of MCI in this pilot study. These findings suggest that self‐administered smartphone applications provide an alternative tool for cognition monitoring and early detection of cognitive impairment.